# The association between prenatal concentrations of polybrominated diphenyl ether and child cognitive and psychomotor function

**DOI:** 10.1097/EE9.0000000000000156

**Published:** 2021-05-11

**Authors:** Giulia Solazzo, Haotian Wu, Hannah E. Laue, Kasey Brennan, Julia M. Knox, Virginie Gillet, Amélie Bovin, Nadia Abdelouahab, Jonathan Posner, Elizabeth Raffanello, Sarah Pieper, Fredrick DuBois Bowman, Daniel Drake, Andrea A. Baccarelli, Larissa Takser

**Affiliations:** a Department of Environmental Health Sciences, Mailman School of Public Health, Columbia University, New York, New York; b Department of Epidemiology, Geisel School of Medicine, Dartmouth College, Lebanon, New Hampshire; c Département de Pédiatrie, Faculté de Médicine et des Sciences de la Santé, Université de Sherbrooke, Quebec, Canada; d Department of Psychiatry, Columbia University Medical Center, and the New York State Psychiatric Institute, New York, New York; e Department of Biostatistics, School of Public Health, University of Michigan, Ann Arbor, Michigan; f Départment de Psychiatrie, Faculté de Médecine et des Sciences de la Santé, Université de Sherbrooke, Sherbrooke, Quebec, Canada

## Abstract

Previous studies suggest a negative association between prenatal polybrominated diphenyl ethers (PBDEs) exposure and child cognitive and psychomotor development. However, the timing of the relationship between PBDE exposure and neurodevelopment is still unclear. We examined the association between PBDE concentration at two different prenatal times (early and late pregnancy) and cognitive function in children 6–8 years of age.

**Methods:**

Eight hundred pregnant women were recruited between 2007 and 2009 from Sherbrooke, Canada. Four PBDE congeners (BDE-47, -99, -100, and -153) were measured in maternal plasma samples collected during early pregnancy (12 weeks of gestation) and at delivery. At 6–8 years of age, 355 children completed a series of subtests spanning multiple neuropsychologic domains: verbal and memory skills were measured using the Wechsler Intelligence Scale for Children, Fourth Edition; visuospatial processing using both Wechsler Intelligence Scale for Children, Fourth Edition and Neuropsychological Assessment second edition; and attention was assessed through the Test of Everyday Attention for Children. Additionally, parents completed subtests from the Developmental Coordination Disorder Questionnaire to measure child motor control. We used linear regression and quantile g-computation models to estimate associations of PBDE congener concentrations and psychologic test scores.

**Results:**

In our models, no significant associations were detected between PBDE mixture and any of the child psychologic scores. BDE-99 concentration at delivery was nominally associated with higher scores on short-term and working memory while a decrease in spatial perception and reasoning was nominally associated with higher BDE-100 concentration at delivery.

**Conclusion:**

Overall, our results did not show a significant association between PBDEs and child cognitive and motor development.

## Abstract 2

Supplemental Digital Content is available in the text.

**What this study adds**

Several studies have analyzed the association between PBDEs and child development. Some suggest that prenatal PBDE concentration may affect cognitive or motor function, but the results vary between cohorts. Our cohort did not show any correlation, but we discussed these differences and brings new questions about why different cohorts reported such different patterns. Additionally, our study considered two different times of pregnancy. These are useful information that can clarify the relationship between prenatal concentration of PBDEs and child development.

## Introduction

Polybrominated diphenyl ethers (PBDEs) are brominated aromatic compounds with two phenyl rings linked by an ether bond. The position and number of the bromine atoms results in 209 possible compounds, referred to as PBDE congeners. Before being banned or phased out in Europe, Canada, and the United States, PBDEs were widely used as flame retardants in a variety of products like clothing, furniture, household goods, electronics, car seats, and more.^1^ While PBDEs are no longer added to newly manufactured products, extant PBDE-containing products are still being used in most households in the United States and worldwide. As PBDEs are not chemically bound to the polymers where they are contained, they can migrate from products into indoor and outdoor environments, resulting in widespread human exposures.^1^

PBDEs are lipophilic, resistant to degradation, and have a tendency to accumulate in sediments and persist in the environment.^2^ Indeed, studies in animal and human models show the propensity of PBDEs to bioaccumulate.^3,4^ In particular, the five congeners most commonly found in human tissue are BDE-47, BDE-99, BDE-100, BDE-153, and BDE-154.^5^ One exposure pathway is through diet, as PBDEs have been detected in fish, poultry, pork, beef, vegetables, and dairy products.^6,7^ PBDEs have also been detected in indoor air and house dust, and hand-to-mouth behaviors among toddlers cause ingestion of PBDE-containing dust, resulting in high body burdens in this sensitive population.^8^ This contamination may occur via volatilization of the lower brominated congeners from furniture, textiles, and electrical appliances.^9–11^

PBDEs have been linked to thyroid disorders, reproductive health effects, and adverse neurodevelopmental outcomes.^12^ Previous studies in cord blood, fetal blood, and placental tissue have demonstrated that PBDEs readily cross the placenta and enter fetal circulation.^13–15^ A recent review^16^ associated PBDE exposure with attention and behavior problems as well as decreased cognitive and motor function.^17–22^ Moreover, these studies also suggest that the impact of prenatal PBDE exposure on child neurodevelopment may affect different domains based on the timing of exposure. For example, studies using the Health Outcomes and Measures of Environment (HOME) cohort reported negative associations between PBDE exposure in early pregnancy (16 weeks of gestation) and child cognitive and behavioral problems.^17,22,23^ Chen et al found that prenatal exposure to BDE-47 is associated with decrements in cognitive abilities and increases in hyperactive behaviors and externalizing behavior problems in children 5 years of age. Vuong et al also reported a poorer behavior regulation in children exposed to PBDEs at early pregnancy. Zhang et al described an increase of externalizing behavior problems associated with prenatal exposure to PBDEs, and an inverse association between prenatal PBDE concentration and reading and cognitive abilities in children 8 years of age. Studies that measured PBDE exposure during late pregnancy (>20 weeks of gestation or delivery) reported adverse associations with a deficit in attention and motor control.^18–20,24^ For example, a study in cord blood plasma showed a negative association between BDE-47 and psychomotor development index at 1 year,^20^ while another study reported an adverse association between prenatal concentration of PBDEs and fine motor coordination in children at 5–7 years of age.^19^

Despite the large number of studies examining the association of prenatal PBDE exposure and child development, none of those studies specifically compared the effects of early versus late pregnancy PBDE exposure on child cognitive and motor function in the same population. Therefore, it is still not clear how the timing of PBDE exposure may differentially affect child neurodevelopment. Thus, in this study, we analyzed the associations of PBDE concentrations during early and late prenatal development with attention, cognitive and motor function in children at 6–8 years of age.

## Material and methods

### Population

Located in Quebec, Canada, the GEStation and Environment cohort enrolled pregnant women at the Research Center of the Centre Hospitalier Universitaire de Sherbrooke between September 2007 and December 2008. As described previously,^25^ 400 women were recruited during their first prenatal care visit at less than 20 weeks of pregnancy while an additional 400 women were enrolled at the time of delivery. Eligibility criteria included maternal age ≥18 years and no known thyroid disease. Women who planned to move out of the region in the following 3 years were excluded. At 6–8 years of age, 355 children completed a series of neurocognitive tests. The study protocol was approved by the Human Research Ethics Committee of the Centre Hospitalier Universitaire de Sherbrooke. Additional analyses were approved by the Columbia University Medical Center IRB. Each participant signed an informed consent agreement.

### Exposure

Maternal blood samples (10 mL) were collected at both early pregnancy (n = 400) and delivery (n = 800) in Sodium/Heparin Vacutainer Hemogard glass tubes (Becton-Dickinson, San Jose, California). Whole blood was separated into components, and the plasma was frozen at −20 °C in decontaminated Supelco glass storage tubes (Supelco, Inc., Bellefonte, Pennsylvania). Maternal plasma concentrations of four PBDE congeners (BDE-47, BDE-99, BDE-100, and BDE-153) were analyzed using solid-phase extraction and gas chromatography spectrometry.^26^ The limit of detection (LOD) was established at 0.0001 ng/mL for each PBDE congeners at delivery, at 0.001 ng/mL for both BDE-99 and BDE-100 in early pregnancy, and at 0.0006 ng/mL for BDE-153 in early pregnancy. Values below the LOD were replaced by LOD divided by the square root of 2. Total lipids (g/L) were measured by the Phillips method.^27^

### Verbal comprehension and memory

To assess verbal comprehension and memory skills, children were asked to complete the Vocabulary, Information, Coding, and Digit Span subtests from the Wechsler Intelligence Scale for Children, Fourth Edition (WISC-IV).^28^ The Vocabulary subtest measures word knowledge and verbal concept formation. The Information subtest requires children to answer general knowledge questions and measures long-term memory and general knowledge. The Coding subtest requires the children to mark rows of shapes with different lines according to a code, which allows for the assessment of fine motor control, short-term memory, and concentration. In the Digit Span subtest, children are asked to repeat a sequence of numbers as heard, and then in reverse order. This subtest measures auditory short-term memory and auditory working memory. All subtest scores were scaled with a mean and SD of 10 ± 3.

### Visuospatial processing

To measure visuospatial processing, children were asked to complete the Design Copying subtest and Trail Making by Developmental Neuropsychological Assessment second edition (NEPSY II).^29^ The NEPSY-II is a comprehensive neuropsychologic test battery that consists of 32 subtests in six functional domains for children 3–16 years of age. The subtest scores scaled by age have a total possible range from 1 to 19. Additionally, the Block Design subtest by WISC-IV was used to measure spatial perception and reasoning. In this subtest, children must put together red-and-white blocks in a pattern according to a displayed model. The score was scaled with a mean and SD of 10 ± 3.

### Attention

To assess attention, children were asked to complete the Sky Search, Score, and Score DT subtests from the Test of Everyday Attention for Children (TEA-Ch).^30^ TEA-Ch is a game-like test intended for use between the ages of six and 16 that measures multiple forms of attention. *Selective attention* is the ability to focus on a specific stimulus. *Sustained attention* is the ability to maintain attention over an extended period. Selective attention was assessed using the “Sky Search” subtest. The Sky Search was adjusted for motor speed, the motor control time-per-target was subtracted from the attention time-per-target. Sustained attention was assessed using “Score” and “Score DT” subtests. All the subtests were adjusted by age.

### Motor control

To measure motor control, parents completed the French Canadian version of the Developmental Coordination Disorder Questionnaire (DCD-Q)^31^ during their child’s follow-up visit. This questionnaire was developed to identify motor problems in children ≥4 years of age. It contains 17 questions related to motor coordination. From these questions, scores were calculated to capture fine motor and handwriting, general coordination, and control during the movement.

### Other information

A research nurse measured the maternal height and weight during the prenatal visit and administered questionnaires to obtain data on age, educational level, marital status, income, obstetrical history, smoking habits, consumption of alcohol, and recreational drug use during pregnancy. Birthweight was extracted from medical records. Gestational age at enrollment and birth were calculated from ultrasonography.

### Statistical analysis

We used quantile-based g-computation (qgcomp) to estimate the overall effect of prenatal PBDE mixture with neurodevelopmental outcomes.^32^ qgcomp is adapted from weighted quantile sum regression, but unlike weighted quantile sum, qgcomp is not constrained to a single direction of effect, does not require splitting of the dataset, and is more robust against unmeasured confounders.^32^ The estimated overall mixture effect can be interpreted as the change in outcome per quantile change in all exposures. The models were fitted using the eponymous gqcomp package (v2.6.0).^32^ To complement the mixture analysis, we used linear regression models to estimate the relationships of PBDEs as individual metabolites with subtest scores. For these models with individual metabolites, we log_2_-transformed all PBDE measurements and a single model with all metabolites was fitted to adjust for co-exposures.

For all models, we used inverse probability weighting (IPW) to address potential selective loss to follow-up and multiple imputations for missing exposure and covariate data.^33^ We created 10 imputed datasets using the “mice” package in R^34^ and within each imputed datasets, calculated stabilized IPW weights for the probability of having 6–8 years of age at visit, then removed those without 6–8 years of age visit and fitted linear or qgcomp models on the resulting dataset with the IPW weights. The estimates from each imputed dataset were then combined using Rubin’s Rule.^35,36^ Due to the two-stage recruitment of our cohort, we analyzed the early pregnancy exposure only among those who were recruited during early pregnancy. For comparison, we also showed the results of complete case analyses in the supplemental tables; http://links.lww.com/EE/A138.

For all analyses, we fit two sets of multivariable models based on previous studies and covariates that showed significant association with any PBDE or any neurodevelopmental outcomes in bivariate models. The minimally adjusted models included maternal age, smoking status during pregnancy, BMI, and total plasma lipids. The fully adjusted models additionally included child sex, child age, birthweight, and gestational age at birth. We chose to present the minimally adjusted results in the main manuscript, but there were no meaningful differences between the results of minimally adjusted and fully adjusted models. Because previous studies have observed sex-specific associations of PBDEs and neurodevelopmental outcomes, we considered effect modification by sex via stratified analyses.

## Results

Relevant demographic information is presented in Table 1. Briefly, we recruited 800 women, 763 delivered and one withdrew from the study. At 6–8 years of age, 355 mother–child pairs completed the neuropsychologic tests. Specifically, of the 386 pairs with available PBDE measurements at early pregnancy, 161 completed the follow up. Of the 560 pairs with available PBDE measurements at late pregnancy, 263 completed the follow-up (Fig. S1; http://links.lww.com/EE/A138). Additional information about the cohort who follow-up the visit are reported in Table S1; http://links.lww.com/EE/A138.

**Table 1.** Characteristics of the GESTE cohort, including all participants with follow-up visits at 6–8 years of age (n = 355).

Characteristics	Mean ± SD or (%)
Maternal characteristics	
Maternal age (years)	36 ± 4.4
Maternal BMI (kg/m^2^)	
1st trimester	24.5 ± 4.9
Delivery	26.3 ±6.0
Educational level (university)	72 (20%)
Consumed alcohol during pregnancy	41 (12%)
Mothers who smoked during pregnancy	28 (8.8%)
Total lipids (g/L)	
1st trimester (n = 161)	6.0 ± 1.5
Delivery (n = 263)	6.9 ± 1.2
Child characteristics	
Child age at testing (years)	6.6 ± 0.5
Birth weight (g)	3408 ± 458
Sex - female	157 (45%)

GESTE indicates GEStation and Environment.

Spearman’s rank correlation tests showed that each BDE was positively correlated with other congeners in early pregnancy and at delivery (Fig. S2; http://links.lww.com/EE/A138). Among the 245 samples with PBDE measurements both in early pregnancy and at delivery, none of the four congeners (BDE-47, BDE-99, BDE-100, and BDE-153) showed a significant correlation between early pregnancy and delivery (*P* > 0.05). Summary PBDE statistics can be found in Table S2; http://links.lww.com/EE/A138.

### Verbal comprehension and memory assessment

**Figure 1.**
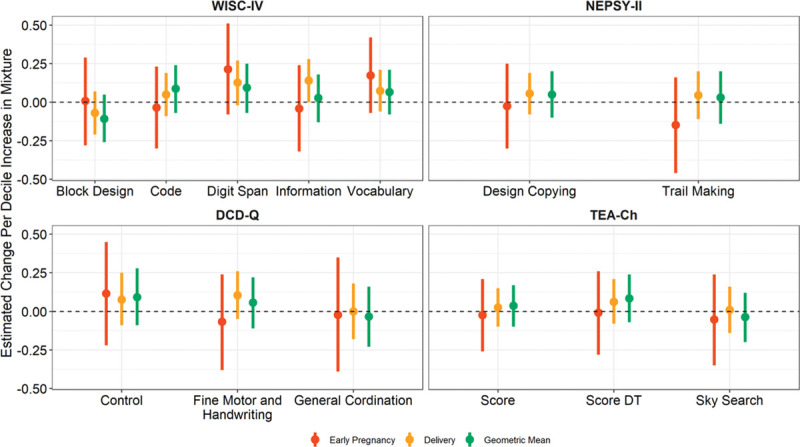
Forest plots showing effect estimates (β) and 95% confidence intervals of the association between child neurodevelopmental measures and plasma PBDE concentrations at early pregnancy, delivery, or their geometric mean. The effect estimates are interpreted as the change in test scores per decile increase in all four congeners (BDE-47, BDE-99, BDE-100, and BDE-153). All models were adjusted for maternal age, smoking status during pregnancy, BMI, and total plasma lipids.

As a mixture, PBDE levels during early pregnancy, delivery, and their average were not associated with vocabulary, information, coding, and digit span subtests of the WISC-IV score (Figure 1 and Table S3; http://links.lww.com/EE/A138). In linear regression models, none of the PBDE levels during early pregnancy was associated with the WISC-IV subtest scores. For congeners measured at delivery, BDE-99 was associated with higher digit span scores (β = 0.14; 95% confidence interval = 0.03, 0.26) (Table S4; http://links.lww.com/EE/A138).

### Visuospatial processing assessment

As a mixture, PBDE levels during early pregnancy, delivery, and their average were not associated with scores from the block design subtest of WISC-IV or Design Copying subtest and Trail Making subtests from NEPSY-II (Figure 1 and Table S3; http://links.lww.com/EE/A138). However, we observed that higher BDE-100 concentration at delivery was significantly associated with a decrease in Block Design score (β = −0.11 per doubling; 95% confidence interval = −0.22, 0) from WISC-IV (Table S4; http://links.lww.com/EE/A138).

### Motor control assessment

As a mixture, PBDE levels during early pregnancy, delivery, and their average were not associated with scores from fine motor and handwriting; general coordination, and control during the movement subtests from DCD-Q (Figure 1 and Table S3; http://links.lww.com/EE/A138). Similarly, no statistically significant associations were observed for the individual metabolite models (Table S4; http://links.lww.com/EE/A138).

### Attention assessment

Sky Search, Score, and Score DT subtests from TEA-Ch did not show any significant association with PBDE mixture at both time, early pregnancy, and delivery (Figure 1 and Table S3; http://links.lww.com/EE/A138). Similarly, no statistically significant associations were observed for the individual metabolite models (Table S4; http://links.lww.com/EE/A138).

### Sex-specific effects

There was a negative association between the average prenatal PBDE, as a mixture, and the TEA-Ch Score subtest, which measures sustained attention, among male children. However, overall, we did not observe general patterns of sex-specific differences (Figure S3; http://links.lww.com/EE/A138).

## Discussion

We did not find any significant associations between PBDE mixture (BDE-47, -99, -100, -153) and any of the assessed child cognitive domains (Verbal Comprehension and Memory, Visuospatial Processing, Motor Control, and Attention). Additionally, when we analyzed the relationships of PBDEs as individual metabolites, we did not observe significant associations with verbal comprehension, motor control, and attention. BDE-99 concentration at delivery was associated with better scores on short-term memory and working memory (Digit Span subtest) while a decrease in spatial perception and reasoning score (Block Design subtest) was associated with higher BDE-100 concentration at delivery, but these may have occurred due to chance and multiple-testing. As such, these metabolite-specific results should be interpreted with caution.

Previous studies have investigated the association of PBDE exposure with verbal and memory skills, and some of them suggested a negative association. A longitudinal cohort recruited in New York showed that a concentration of BDE-100 in cord blood was negatively associated with Verbal IQ scores in children at 36 months.^20^ Similarly, BDE-100 measured in maternal plasma at 16 weeks of gestation was negatively associated with Reading Composite scores in children at 8 years of age.^22^ Also, other PBDE congeners (i.e., BDE-153 and BDE-99) have been associated with deficiencies in language development in children.^18,22^ In contrast with these studies, we did not observe any negative association between child verbal and memory skills. Our results may be inconsistent with previous studies due to differences in the cohorts’ sociodemographic makeup and exposure levels. For example, while GEStation and Environment families are largely upper-middle class, the CHAMACOS cohort only included families that qualified for low-income health insurance.^19^ Our cohort had lower levels of PBDEs compared to the CHAMACOS cohort and World Trade Center cohort^19,20^ and it is unclear whether this difference in exposure level could have contributed to the disparities in results. Additionally, our study and each of the related studies all tested children at different ages (from a few months to 8 years of age).^20,22^

Consistent with a recent study^37^ of 199 children from Ohio, we found no association between prenatal PBDE mixture levels and visuospatial abilities. In contrast, several previous animal studies have reported an association between prenatal PBDE exposure and deficits in visual-spatial learning and memory.^38–40^ We did find that one congener, BDE-100, measured during late pregnancy was negatively associated with child spatial perception at nominal significance. Thus, the overall evidence is mixed, and further investigations are necessary.

It is still unclear if PBDE exposure may affect child motor control. Most studies have not detected significant associations,^16–18,24,41^ and similarly, our study is in line with that research. However, two studies suggested a negative association between motor development and PBDE exposure.^19,20^ Eskenazi et al^19^ found that maternal PBDEs were significantly associated with poor fine motor control at 5–7 years of age, while Herbstman et al^20^ described an association between BDE-100 concentration and lower motor development in 1-year children.

The association between child attention and PBDE exposure has been well investigated.^16^ Previous animal studies suggested that PBDE exposure can affect sustained attention and can increase hyperactivity.^42,43^ Similarly, studies in children have detected correlations between PBDE exposure and child attention deficits. For example, Sagiv et al^24^ described a decrease in attention in children with higher prenatal exposure to PBDEs. Another study described an association between cord blood concentration of both BDE-47 and BDE-153 with attention problems in children at 4 years of age.^44^ However, our results are not consistent with these previous studies; indeed, we did not observe any significant association between prenatal PBDE exposure and child attention. Similar to our findings for verbal and memory domains, the difference could be a consequence of sociodemographic characteristics of our cohort or the use of subtests from TEA-Ch and not the whole test.

A previous study detected a higher PBDE concentration in placental tissues from males compared to females, and they suggested that this different prenatal exposure to PBDE may alter thyroid hormone (TH) function in a sex-specific manner.^45^ Some studies reported that increases in PBDE concentrations were associated with poor executive function, behavior problems, and verbal comprehension in male children,^23,46^ while other studies did not found any significant difference.^24,37,44^ We did not detect any meaningful patterns of sex-specific differences in our cohort. While we did find a negative association between the prenatal PBDE mixture and the Score subtest of TEA-Ch, which measures sustained attention, in male children, this is not supported by the evidence from Score DT subtest, which measures sustained attention under dual tasks.

Although our study did not suggest any association between prenatal exposure to PBDE and child cognitive and motor skills, the overall epidemiologic evidence is mixed. Experimental studies in animals found that prenatal and postnatal exposure to PBDEs may cause long-lasting brain alterations, especially in the domains of motor activity and cognitive behavior.^38–40,42,43^ It is unclear whether PBDEs could directly or indirectly affect brain development. For example, PBDEs may affect neurodevelopment by altering TH homeostasis.^5^ THs are essential for the normal development of the central nervous system. Hypothyroidism has been associated with a large number of neuroanatomical and behavioral effects.^47,48^

The strength of our study is that it measured PBDEs at two different time points during pregnancy. We also considered the effect of PBDE mixture and the effect of each metabolite (BDE-47, -99, -100, and -153). Additionally, the use of WISC-IV, NEPSY-II, TEA-Ch, and DCD-Q subtests to capture the outcomes provides an objective assessment of neurodevelopment. Overall, our population was highly educated and homogenous, but similar to the general Canadian population in terms of PBDE exposure. While limiting generalizability, the relative homogeneity of our cohort may represent an advantage for testing the effects of environmental exposure, because it reduces confounding from hard-to-control confounders such as lifestyle and socioeconomic factors. Another limitation of our study is the lack of pregestational weight. In our models, we adjusted for maternal BMI at the time of the prenatal visit, which reflects both gestational weight gain and pregestational weight but having prepregnancy maternal weight data would have allowed us to assess the influence of both independently. Finally, another limitation of our study is the relatively small sample size.

In conclusion, our results did not provide evidence that prenatal maternal plasma concentrations of PBDEs during either early or late pregnancy are associated with child cognitive and psychomotor domains. There is some suggestive evidence that BDE-100 during late pregnancy is associated with child spatial perception, which is consistent with evidence from animal studies, but this should be interpreted cautiously without additional replication. The inconsistency between our results and those of previous studies may be due to differences in population characteristics (e.g., child age) and exposure levels, suggesting that future studies should consider factors such as social stress and demographic differences in the study of child cognitive and motor developmental effects of environmental chemicals.

## Conflicts of interest statement

The authors declare that they have no conflicts of interest with regard to the content of this report.

## Supplementary Material


