# Supplementary material for: The association between prenatal concentrations of polybrominated diphenyl ether and child cognitive and psychomotor function
Source: Environ Epidemiol. 2021 May 11;5(3):e156. doi: 10.1097/EE9.0000000000000156 (PMC8196085; doi:10.1097/EE9.0000000000000156)
Supplement: Supplementary file 1 [file ee9-5-e156-s001.docx]

**The association between prenatal concentrations of polybrominated diphenyl ether (PBDEs) at pregnancy and child cognitive and psychomotor development.**

**Supplemental Tables and Figures**

**Table S1.** Demographic and PBDE exposure comparison between the group who follow-up the visit and the group who did not follow-up the visit

**Table S2.** Distribution of uncorrected polybrominated diphenyl ethers (PBDEs) in the GESTE Study Population

**Table S3.** Model estimates for the association between PBDE mixture and subtest scores from Quantile G-Computation Models.

**Table S4.** Linear regression model estimates for the association between log2-transformed PBDE metabolites and subtest scores.

**Figure S1.** Graphic representation of cohort and recruitment process. R1 = First Recruitment; R2 = Second Recruitment; R3 = Child follow-up; T1= First Recruitment Period; T2 = Second Recruitment Period.

**Figure S2.** Spearman’s rank correlation between each PBDE (BDE-47, BDE-99, BDE-100, and BDE-153) in early pregnancy (A) and between each PBDE (BDE-47, BDE-99, BDE-100, and BDE-153) at delivery (B).

**Figure S3.** Forest plots showing effect estimates (β) and 95% confidence intervals of the association between child neurodevelopmental measures and plasma PBDE concentrations at early pregnancy, delivery, or their geometric mean, stratified by A) Male and B) Female children. The effect estimates are interpreted as the change in test scores per decile increase in all four congeners (BDE-47, BDE-99, BDE-100, and BDE-153). All models were adjusted for maternal age, smoking status during pregnancy, BMI, and total plasma lipids.

| **Table S1**. Demographic and PBDE exposure comparison between the group who follow-up the visit and the group who did not follow-up the visit | | | |
| --- | --- | --- | --- |
|  | Children with follow-up visit (n=365) | Children with no follow-up visit (n=407) | p-value* |
| Maternal age (years) | 28.87 +/- 4.41 (19-40; n=362) (95% CI=28.42-29.33) | 28.03 +/- 4.74 (18-43; n=400)  (95% CI= 27.57-28.49) | 0.01 |
| Gestational age (weeks) | 39.14 +/- 1.4 (33-41; n=362) | 38.93 +/- 1.59 (32-41; n=399) | 0.07 |
| Consumed alcohol during pregnancy (%) | 24.86 (n=354) | 19.22 (n=359) | 0.07 |
| Mothers who smoked during pregnancy (%) | 7.91 (n=354) | 23.12 (n=359) | <.0001 |
| BMI at 1st Trimester | 26.17 +/- 5.99 (17.10-48.23; n=181) | 25.01 +/- 5.46 (15.82-48.99; n=214) | 0.07 |
| BMI recruited at Delivery** | 25.75 +/- 6.25 (17.32-55.60; n=177) | 25.85 +/- 6.10 (16.44-52.33; n=185) | 0.86 |
| Birth weight (gr) | 3397.56 +/- 486.20 (1785-4910; n=365) | 3341.06 +/- 508.92 (1160.00 - 4740.00$; n=406) | 0.11 |
| Child age (% female) | 45.48 (n=365) | 47.42 (n=407) | 0.59 |
| Mother education level | (n=359) | (n=375) | <.0001 |
| University (%) | 38.72 | 24.53 |  |
| High school (%) | 27.86 | 27.2 |  |
| Middle school (%) | 11.98 | 24.27 |  |
| Elementary school (%) | 1.67 | 2.13 |  |
| Others (%) | 19.78 | 21.87 |  |
| Income ($) | 70701 +/- 41349 (2600 –450000 $; n=342) | 60328 +/- 37690 (8400 – 500000 $; n=325) | 0.0003 |
| EXPOSURE INFORMATION |  |  |  |
| PBDE-47 (1st Trimester) | 0.205 +/- 0.243 (0.006-1.679; n=161) | 0.203 +/- 0.300 (0.007-2.735; n=189) | 0.95 |
| PBDE-99 (1st Trimester) | 0.029 +/- 0.040 (0.001-0.250; n=161) | 0.031 +/- 0.058 (0.001-0.48; n=190) | 0.6 |
| PBDE-100 (1st Trimester) | 0.019 +/- 0.052 (0.001-0.365; n=161) | 0.015 +/- 0.033 (0.001-0.269; n=190) | 0.37 |
| PBDE-153 (1st Trimester) | 0.039 +/- 0.068 (0.0003-0.345; n=161 | 0.039 +/- 0.070 (0.0003-0.456; n=190) | 0.93 |
| PBDE-47 (Delivery) | 0.128 +/- 0.313 (0.0001-4.327; n=267) | 0.127 +/- 0.197 (0.0001-1.656; n=286) | 0.96 |
| PBDE-99 (Delivery) | 0.067 +/- 0.219 (0.00004-3.302; n=267) | 0.056 +/- 0.096 (0.00003-1.029; n=286) | 0.43 |
| PBDE-100 (Delivery) | 0.062 +/- 0.123 (0.0001-1.268; n=267) | 0.056 +/- 0.087 (0.0001-0.643; n=286) | 0.56 |
| PBDE-153 (Delivery) | 0.112 +/- 0.641 (0.0001-10.368; n=267) | 0.074 +/- 0.117 (0.0001-1.059; n=286) | 0.35 |

*p-value for categorical variables were obtained with chi-square test, while p-value fir quantitative variables were obtained with t-test.

**The BMI for these women was measured before pregnancy

| **Table S2**. Distribution of uncorrected polybrominated diphenyl ethers (PBDEs) in the GESTE Study Population | | | | |
| --- | --- | --- | --- | --- |
| **PBDE** | **Early Pregnancy (N=386)** | **Delivery (N=560)** | **Spearman Correlation (N=245)** | |
|  | **Mean (ng/mL) ± SD** | **Mean (ng/mL) ± SD** | **Rho** | **p-value** |
| BDE-47 | 0.201 ±0.26 | 0.128 ±0.26 | 0.052 | 0.42 |
| BDE-99 | 0.029 ±0.05 | 0.062 ±0.17 | -0.077 | 0.23 |
| BDE-100 | 0.016 ±0.04 | 0.059 ±0.11 | 0.038 | 0.56 |
| BDE-153 | 0.039 ±0.07 | 0.092 ±0.45 | 0.114 | 0.08 |

| **Table S3. Model estimates for the association between PBDE mixture and subtest scores from Quantile G-Computation Models.** | | | | | | | | | | | | | |
| --- | --- | --- | --- | --- | --- | --- | --- | --- | --- | --- | --- | --- | --- |
|  |  | **Early Pregnancy** | | | | **Delivery** | | | | **Geometric Mean** | | | |
| **Test** | **Subtest** | **N** | **β** | **95% CI** | **p-value** | **N** | **β** | **95% CI** | **p-value** | **N** | **β** | **95% CI** | **p-value** |
| **Model 1 (Minimally Adjusted)*** | | | | | | | | | | | | | |
| WISC | Vocabulary | 165 | 0.17 | -0.07, 0.42 | 0.17 | 355 | 0.07 | -0.06, 0.21 | 0.28 | 355 | 0.07 | -0.08, 0.21 | 0.38 |
|  | Code | 165 | -0.04 | -0.3, 0.23 | 0.79 | 355 | 0.05 | -0.09, 0.19 | 0.49 | 355 | 0.09 | -0.07, 0.24 | 0.26 |
|  | Digit Span | 165 | 0.21 | -0.08, 0.51 | 0.16 | 355 | 0.13 | -0.02, 0.27 | 0.09 | 355 | 0.09 | -0.07, 0.25 | 0.25 |
|  | Information | 165 | -0.04 | -0.32, 0.24 | 0.77 | 355 | 0.14 | 0, 0.28 | 0.05 | 355 | 0.03 | -0.13, 0.18 | 0.73 |
|  | Block Design | 165 | 0.01 | -0.28, 0.29 | 0.96 | 355 | -0.07 | -0.21, 0.07 | 0.34 | 355 | -0.11 | -0.26, 0.05 | 0.18 |
| NEPSY-II | Design Copying | 165 | -0.03 | -0.3, 0.25 | 0.86 | 355 | 0.05 | -0.08, 0.19 | 0.42 | 355 | 0.05 | -0.1, 0.2 | 0.51 |
|  | Trail Making | 165 | -0.15 | -0.46, 0.16 | 0.35 | 355 | 0.04 | -0.11, 0.2 | 0.58 | 355 | 0.03 | -0.14, 0.2 | 0.74 |
| DCD-Q | Total Score | 165 | 0.03 | -0.84, 0.89 | 0.95 | 355 | 0.18 | -0.24, 0.6 | 0.41 | 355 | 0.11 | -0.35, 0.57 | 0.62 |
|  | General Coordination | 165 | -0.02 | -0.39, 0.35 | 0.90 | 355 | 0.00 | -0.18, 0.18 | 0.99 | 355 | -0.03 | -0.23, 0.16 | 0.73 |
|  | Fine Motor and Handwriting | 165 | -0.07 | -0.38, 0.24 | 0.67 | 355 | 0.10 | -0.05, 0.26 | 0.18 | 355 | 0.06 | -0.11, 0.22 | 0.50 |
|  | Control | 165 | 0.12 | -0.22, 0.45 | 0.50 | 355 | 0.08 | -0.09, 0.25 | 0.38 | 355 | 0.09 | -0.09, 0.28 | 0.33 |
| TEA-Ch | Score | 165 | -0.03 | -0.26, 0.21 | 0.84 | 355 | 0.02 | -0.1, 0.15 | 0.69 | 355 | 0.04 | -0.1, 0.17 | 0.61 |
|  | Score DT | 165 | -0.01 | -0.28, 0.26 | 0.95 | 355 | 0.06 | -0.08, 0.21 | 0.40 | 355 | 0.08 | -0.07, 0.24 | 0.30 |
|  | Sky Search | 165 | -0.05 | -0.35, 0.24 | 0.72 | 355 | 0.01 | -0.14, 0.16 | 0.91 | 355 | -0.04 | -0.2, 0.12 | 0.65 |
| **Model 2 (Fully Adjusted)**** | | | | | | | | | | | | | |
| WISC | Vocabulary | 165 | 0.17 | -0.08, 0.42 | 0.18 | 355 | 0.07 | -0.06, 0.2 | 0.30 | 355 | 0.06 | -0.08, 0.21 | 0.38 |
|  | Code | 165 | -0.02 | -0.27, 0.23 | 0.88 | 355 | 0.07 | -0.07, 0.2 | 0.35 | 355 | 0.11 | -0.04, 0.26 | 0.14 |
|  | Digit Span | 165 | 0.13 | -0.16, 0.43 | 0.38 | 355 | 0.13 | -0.01, 0.27 | 0.08 | 355 | 0.09 | -0.06, 0.25 | 0.23 |
|  | Information | 165 | -0.06 | -0.32, 0.2 | 0.65 | 355 | 0.15 | 0.02, 0.28 | 0.02 | 355 | 0.04 | -0.1, 0.18 | 0.60 |
|  | Block Design | 165 | 0.03 | -0.24, 0.31 | 0.80 | 355 | -0.04 | -0.17, 0.1 | 0.57 | 355 | -0.08 | -0.23, 0.07 | 0.29 |
| NEPSY-II | Design Copying | 165 | 0.04 | -0.24, 0.31 | 0.80 | 355 | 0.03 | -0.1, 0.16 | 0.62 | 355 | 0.04 | -0.1, 0.18 | 0.59 |
|  | Trail Making | 165 | -0.16 | -0.48, 0.15 | 0.30 | 355 | 0.04 | -0.11, 0.2 | 0.58 | 355 | 0.04 | -0.13, 0.21 | 0.67 |
| DCD-Q | Total Score | 165 | 0.10 | -0.79, 0.98 | 0.83 | 355 | 0.18 | -0.24, 0.6 | 0.40 | 355 | 0.14 | -0.32, 0.59 | 0.55 |
|  | General Coordination | 165 | -0.01 | -0.39, 0.37 | 0.96 | 355 | 0.01 | -0.17, 0.19 | 0.95 | 355 | -0.02 | -0.21, 0.17 | 0.84 |
|  | Fine Motor and Handwriting | 165 | -0.04 | -0.35, 0.26 | 0.78 | 355 | 0.09 | -0.06, 0.23 | 0.24 | 355 | 0.05 | -0.11, 0.21 | 0.52 |
|  | Control | 165 | 0.15 | -0.19, 0.49 | 0.39 | 355 | 0.09 | -0.08, 0.26 | 0.31 | 355 | 0.11 | -0.08, 0.29 | 0.25 |
| TEA-Ch | Score | 165 | -0.04 | -0.28, 0.2 | 0.73 | 355 | 0.02 | -0.1, 0.15 | 0.73 | 355 | 0.04 | -0.1, 0.17 | 0.58 |
|  | Score DT | 165 | 0.00 | -0.27, 0.28 | 0.98 | 355 | 0.05 | -0.09, 0.2 | 0.50 | 355 | 0.07 | -0.09, 0.23 | 0.38 |
|  | Sky Search | 165 | -0.10 | -0.4, 0.2 | 0.50 | 355 | 0.02 | -0.13, 0.17 | 0.78 | 355 | -0.03 | -0.19, 0.13 | 0.70 |
| *Adjusted for maternal age, smoking status during pregnancy, BMI, and plasma total lipids.  **Adjusted for maternal age, smoking status during pregnancy, BMI, child age, child sex, gestational age at birth, birthweight, and plasma total lipids | | | | | | | | | | | | | |

| **Table S4. Linear regression model estimates for the association between log2-transformed PBDE metabolites and subtest scores.** | | | | | | | | | | | | | | |
| --- | --- | --- | --- | --- | --- | --- | --- | --- | --- | --- | --- | --- | --- | --- |
| **Early Pregnancy - Complete Cases** | | | | | | | | | | | | | | |
|  |  |  | **BDE-47** | | | **BDE-99** | | | **BDE-100** | | | **BDE-153** | | |
| **Test** | **Outcome** | **N** | **β** | **95% CI** | **p-value** | **β** | **95% CI** | **p-value** | **β** | **95% CI** | **p-value** | **β** | **95% CI** | **p-value** |
| WISC | Vocabulary | 152 | -0.27 | -0.57, 0.04 | 0.08 | 0.05 | -0.23, 0.33 | 0.71 | -0.02 | -0.22, 0.18 | 0.86 | 0.13 | -0.03, 0.28 | 0.10 |
|  | Code | 152 | -0.04 | -0.34, 0.27 | 0.82 | 0.22 | -0.07, 0.5 | 0.13 | -0.27 | -0.47, -0.07 | 0.01 | 0.02 | -0.14, 0.18 | 0.81 |
|  | Digit Span | 152 | -0.11 | -0.45, 0.23 | 0.52 | 0.06 | -0.26, 0.37 | 0.73 | -0.06 | -0.28, 0.16 | 0.58 | 0.05 | -0.12, 0.23 | 0.55 |
|  | Information | 152 | -0.09 | -0.44, 0.26 | 0.60 | -0.01 | -0.34, 0.31 | 0.93 | -0.14 | -0.37, 0.09 | 0.22 | -0.05 | -0.23, 0.13 | 0.57 |
|  | Block Design | 152 | -0.08 | -0.41, 0.26 | 0.65 | 0.00 | -0.31, 0.31 | 0.98 | -0.08 | -0.3, 0.14 | 0.46 | 0.09 | -0.08, 0.26 | 0.30 |
| NEPSY-II | Design Copying | 152 | -0.24 | -0.57, 0.09 | 0.16 | 0.02 | -0.28, 0.33 | 0.88 | -0.10 | -0.31, 0.12 | 0.37 | 0.08 | -0.09, 0.24 | 0.38 |
|  | Trail Making | 152 | -0.05 | -0.42, 0.32 | 0.78 | 0.01 | -0.33, 0.35 | 0.97 | -0.19 | -0.43, 0.05 | 0.13 | -0.02 | -0.21, 0.17 | 0.87 |
| DCD-Q | Total Score | 152 | 0.35 | -0.7, 1.4 | 0.51 | -0.78 | -1.75, 0.2 | 0.12 | 0.37 | -0.31, 1.06 | 0.28 | 0.10 | -0.43, 0.64 | 0.71 |
|  | General Coordination | 152 | 0.25 | -0.2, 0.69 | 0.27 | -0.28 | -0.69, 0.13 | 0.18 | 0.08 | -0.2, 0.37 | 0.56 | -0.02 | -0.25, 0.2 | 0.85 |
|  | Fine Motor and Handwriting | 152 | 0.06 | -0.31, 0.43 | 0.74 | -0.23 | -0.58, 0.11 | 0.18 | 0.15 | -0.09, 0.39 | 0.21 | 0.05 | -0.14, 0.24 | 0.58 |
|  | Control | 152 | 0.04 | -0.37, 0.46 | 0.84 | -0.27 | -0.65, 0.12 | 0.17 | 0.13 | -0.14, 0.4 | 0.33 | 0.07 | -0.14, 0.28 | 0.51 |
| TEA-Ch | Score | 151 | 0.11 | -0.18, 0.39 | 0.47 | -0.15 | -0.42, 0.11 | 0.25 | -0.06 | -0.25, 0.13 | 0.54 | 0.06 | -0.08, 0.21 | 0.39 |
|  | Score DT | 145 | -0.03 | -0.36, 0.31 | 0.87 | -0.19 | -0.5, 0.12 | 0.22 | 0.07 | -0.15, 0.29 | 0.55 | 0.06 | -0.11, 0.23 | 0.49 |
|  | Sky Search | 150 | -0.12 | -0.48, 0.24 | 0.52 | 0.03 | -0.31, 0.36 | 0.87 | 0.08 | -0.16, 0.31 | 0.51 | -0.12 | -0.3, 0.07 | 0.21 |
| **Early Pregnancy - MI + IPW** | | | | | | | | | | | | | | |
|  |  |  | **BDE-47** | | | **BDE-99** | | | **BDE-100** | | | **BDE-153** | | |
| **Test** | **Outcome** | **N** | **β** | **95% CI** | **p-value** | **β** | **95% CI** | **p-value** | **β** | **95% CI** | **p-value** | **β** | **95% CI** | **p-value** |
| WISC | Vocabulary | 165 | -0.17 | -0.46, 0.12 | 0.26 | 0.07 | -0.14, 0.29 | 0.50 | 0.08 | -0.15, 0.32 | 0.49 | 0.11 | -0.05, 0.26 | 0.17 |
|  | Code | 165 | 0.14 | -0.25, 0.54 | 0.48 | 0.26 | -0.09, 0.61 | 0.14 | -0.16 | -0.4, 0.07 | 0.18 | 0.04 | -0.15, 0.23 | 0.70 |
|  | Digit Span | 165 | 0.05 | -0.46, 0.56 | 0.85 | 0.18 | -0.22, 0.58 | 0.38 | -0.11 | -0.4, 0.19 | 0.48 | 0.14 | -0.05, 0.34 | 0.15 |
|  | Information | 165 | -0.09 | -0.44, 0.26 | 0.61 | 0.00 | -0.25, 0.26 | 0.98 | -0.10 | -0.31, 0.12 | 0.39 | -0.03 | -0.21, 0.14 | 0.70 |
|  | Block Design | 165 | 0.07 | -0.33, 0.46 | 0.75 | 0.17 | -0.13, 0.48 | 0.26 | -0.07 | -0.37, 0.23 | 0.65 | 0.15 | -0.09, 0.39 | 0.23 |
| NEPSY-II | Design Copying | 165 | -0.09 | -0.46, 0.27 | 0.62 | 0.14 | -0.14, 0.41 | 0.33 | -0.13 | -0.37, 0.1 | 0.27 | 0.14 | -0.04, 0.31 | 0.12 |
|  | Trail Making | 165 | -0.09 | -0.58, 0.41 | 0.73 | 0.33 | 0.01, 0.66 | 0.05 | -0.28 | -0.56, -0.01 | 0.04 | 0.10 | -0.12, 0.32 | 0.38 |
| DCD-Q | Total Score | 165 | 0.00 | -0.98, 0.99 | 1.00 | -0.21 | -1.13, 0.71 | 0.65 | 0.05 | -0.75, 0.85 | 0.90 | 0.09 | -0.5, 0.69 | 0.76 |
|  | General Coordination | 165 | 0.03 | -0.5, 0.56 | 0.91 | -0.16 | -0.64, 0.32 | 0.52 | -0.05 | -0.4, 0.31 | 0.79 | 0.00 | -0.25, 0.25 | 0.98 |
|  | Fine Motor and Handwriting | 165 | -0.01 | -0.35, 0.33 | 0.95 | 0.09 | -0.23, 0.41 | 0.59 | -0.01 | -0.28, 0.26 | 0.96 | 0.04 | -0.17, 0.24 | 0.71 |
|  | Control | 165 | -0.02 | -0.39, 0.36 | 0.93 | -0.14 | -0.63, 0.34 | 0.57 | 0.11 | -0.24, 0.46 | 0.54 | 0.06 | -0.18, 0.29 | 0.64 |
| TEA-Ch | Score | 165 | -0.03 | -0.37, 0.32 | 0.88 | -0.09 | -0.48, 0.3 | 0.64 | -0.11 | -0.41, 0.2 | 0.49 | -0.04 | -0.25, 0.17 | 0.72 |
|  | Score DT | 165 | -0.05 | -0.37, 0.26 | 0.74 | 0.01 | -0.24, 0.25 | 0.95 | 0.02 | -0.21, 0.24 | 0.89 | 0.07 | -0.08, 0.22 | 0.34 |
|  | Sky Search | 165 | -0.12 | -0.59, 0.35 | 0.61 | -0.14 | -0.48, 0.2 | 0.42 | 0.07 | -0.22, 0.36 | 0.63 | -0.12 | -0.3, 0.06 | 0.19 |
| **Delivery - Complete Cases** | | | | | | | | | | | | | | |
|  |  |  | **BDE-47** | | | **BDE-99** | | | **BDE-100** | | | **BDE-153** | | |
| **Test** | **Outcome** | **N** | **β** | **95% CI** | **p-value** | **β** | **95% CI** | **p-value** | **β** | **95% CI** | **p-value** | **β** | **95% CI** | **p-value** |
| WISC | Vocabulary | 237 | 0.15 | -0.08, 0.38 | 0.21 | 0.00 | -0.13, 0.12 | 0.99 | -0.15 | -0.3, 0.01 | 0.06 | 0.12 | 0, 0.24 | 0.04 |
|  | Code | 238 | -0.14 | -0.38, 0.1 | 0.25 | 0.05 | -0.08, 0.18 | 0.45 | 0.04 | -0.13, 0.2 | 0.67 | 0.08 | -0.04, 0.2 | 0.20 |
|  | Digit Span | 238 | 0.05 | -0.21, 0.31 | 0.73 | 0.12 | -0.02, 0.26 | 0.09 | -0.05 | -0.23, 0.12 | 0.54 | -0.04 | -0.17, 0.09 | 0.58 |
|  | Information | 238 | 0.11 | -0.15, 0.37 | 0.40 | 0.06 | -0.08, 0.21 | 0.38 | -0.05 | -0.23, 0.12 | 0.56 | 0.03 | -0.1, 0.16 | 0.65 |
|  | Block Design | 238 | -0.01 | -0.27, 0.25 | 0.95 | 0.07 | -0.07, 0.21 | 0.33 | -0.15 | -0.32, 0.02 | 0.09 | 0.01 | -0.12, 0.14 | 0.89 |
| NEPSY-II | Design Copying | 238 | 0.08 | -0.16, 0.33 | 0.52 | 0.07 | -0.06, 0.2 | 0.31 | -0.10 | -0.26, 0.06 | 0.23 | 0.02 | -0.1, 0.14 | 0.74 |
|  | Trail Making | 238 | -0.05 | -0.33, 0.23 | 0.71 | 0.11 | -0.05, 0.26 | 0.17 | 0.04 | -0.14, 0.23 | 0.66 | -0.03 | -0.17, 0.11 | 0.65 |
| DCD-Q | Total Score | 238 | 0.01 | -0.75, 0.77 | 0.98 | 0.17 | -0.24, 0.58 | 0.42 | -0.12 | -0.62, 0.38 | 0.64 | 0.05 | -0.33, 0.43 | 0.79 |
|  | General Coordination | 238 | -0.04 | -0.36, 0.27 | 0.79 | 0.05 | -0.12, 0.22 | 0.58 | 0.05 | -0.15, 0.26 | 0.61 | -0.08 | -0.24, 0.07 | 0.30 |
|  | Fine Motor and Handwriting | 238 | 0.11 | -0.16, 0.37 | 0.42 | 0.04 | -0.1, 0.19 | 0.54 | -0.08 | -0.26, 0.09 | 0.36 | 0.04 | -0.1, 0.17 | 0.60 |
|  | Control | 238 | -0.05 | -0.36, 0.25 | 0.73 | 0.08 | -0.09, 0.24 | 0.38 | -0.09 | -0.3, 0.11 | 0.37 | 0.10 | -0.06, 0.26 | 0.21 |
| TEA-Ch | Score | 235 | -0.02 | -0.23, 0.2 | 0.87 | 0.02 | -0.09, 0.14 | 0.71 | 0.00 | -0.14, 0.14 | 0.98 | 0.02 | -0.08, 0.13 | 0.68 |
|  | Score DT | 228 | 0.10 | -0.16, 0.37 | 0.45 | 0.01 | -0.13, 0.16 | 0.88 | -0.16 | -0.33, 0.01 | 0.07 | 0.12 | -0.01, 0.25 | 0.07 |
|  | Sky Search | 235 | 0.07 | -0.19, 0.33 | 0.59 | -0.04 | -0.18, 0.1 | 0.58 | 0.05 | -0.12, 0.22 | 0.59 | -0.02 | -0.14, 0.11 | 0.81 |
| **Delivery - MI + IPW** | | | | | | | | | | | | | | |
|  |  |  | **BDE-47** | | | **BDE-99** | | | **BDE-100** | | | **BDE-153** | | |
| **Test** | **Outcome** | **N** | **β** | **95% CI** | **p-value** | **β** | **95% CI** | **p-value** | **β** | **95% CI** | **p-value** | **β** | **95% CI** | **p-value** |
| WISC | Vocabulary | 355 | 0.10 | -0.04, 0.25 | 0.16 | 0.02 | -0.08, 0.12 | 0.72 | -0.02 | -0.13, 0.09 | 0.70 | 0.06 | -0.03, 0.14 | 0.17 |
|  | Code | 355 | 0.03 | -0.15, 0.21 | 0.75 | 0.07 | -0.03, 0.17 | 0.15 | 0.06 | -0.05, 0.16 | 0.29 | 0.05 | -0.04, 0.13 | 0.28 |
|  | Digit Span | 355 | 0.11 | -0.07, 0.29 | 0.22 | 0.14 | 0.03, 0.26 | 0.02 | 0.03 | -0.06, 0.13 | 0.48 | -0.03 | -0.11, 0.05 | 0.52 |
|  | Information | 355 | 0.14 | -0.04, 0.31 | 0.12 | 0.06 | -0.04, 0.16 | 0.22 | 0.03 | -0.07, 0.13 | 0.58 | 0.02 | -0.08, 0.11 | 0.74 |
|  | Block Design | 355 | -0.08 | -0.3, 0.13 | 0.44 | 0.00 | -0.1, 0.11 | 0.93 | -0.11 | -0.22, 0 | 0.05 | -0.06 | -0.15, 0.04 | 0.23 |
| NEPSY-II | Design Copying | 355 | 0.02 | -0.13, 0.17 | 0.81 | 0.06 | -0.03, 0.15 | 0.18 | -0.01 | -0.12, 0.1 | 0.90 | 0.00 | -0.08, 0.08 | 0.99 |
|  | Trail Making | 355 | 0.01 | -0.16, 0.18 | 0.89 | 0.08 | -0.02, 0.18 | 0.12 | 0.06 | -0.06, 0.18 | 0.36 | -0.03 | -0.13, 0.07 | 0.56 |
| DCD-Q | Total Score | 355 | 0.23 | -0.24, 0.71 | 0.33 | 0.14 | -0.14, 0.42 | 0.33 | 0.06 | -0.21, 0.33 | 0.66 | 0.02 | -0.24, 0.28 | 0.87 |
|  | General Coordination | 355 | 0.03 | -0.19, 0.26 | 0.76 | 0.05 | -0.07, 0.16 | 0.43 | 0.05 | -0.08, 0.17 | 0.46 | -0.05 | -0.16, 0.05 | 0.34 |
|  | Fine Motor and Handwriting | 355 | 0.13 | -0.04, 0.29 | 0.14 | 0.05 | -0.06, 0.17 | 0.34 | 0.02 | -0.08, 0.13 | 0.66 | 0.01 | -0.09, 0.11 | 0.82 |
|  | Control | 355 | 0.07 | -0.09, 0.24 | 0.39 | 0.04 | -0.07, 0.15 | 0.50 | -0.01 | -0.1, 0.09 | 0.85 | 0.06 | -0.03, 0.16 | 0.21 |
| TEA-Ch | Score | 355 | 0.04 | -0.09, 0.17 | 0.55 | 0.04 | -0.05, 0.12 | 0.40 | 0.02 | -0.08, 0.11 | 0.74 | -0.02 | -0.09, 0.06 | 0.70 |
|  | Score DT | 355 | 0.01 | -0.13, 0.15 | 0.93 | 0.00 | -0.1, 0.1 | 1.00 | -0.06 | -0.15, 0.03 | 0.19 | 0.03 | -0.07, 0.12 | 0.58 |
|  | Sky Search | 355 | 0.01 | -0.16, 0.17 | 0.95 | -0.05 | -0.17, 0.08 | 0.46 | 0.00 | -0.11, 0.12 | 0.95 | 0.03 | -0.06, 0.12 | 0.50 |
| All models adjusted for maternal smoking status and BMI during pregnancy, plasma total lipids, and co-exposures. | | | | | | | | | | | | | | |

**Figure S1.** Graphic representation of cohort and recruitment process. R1 = First Recruitment; R2 = Second Recruitment; R3 = Child follow-up; T1= First Recruitment Period; T2 = Second Recruitment Period.

**
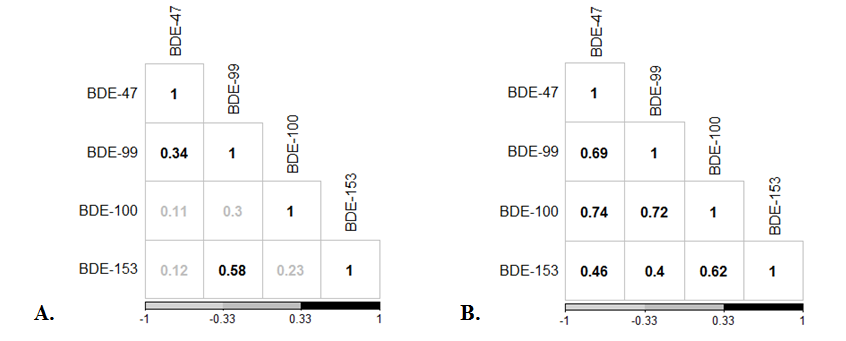
**

**Figure S2.** Spearman’s rank correlation between each PBDE (BDE-47, BDE-99, BDE-100, and BDE-153) in early pregnancy (A) and between each PBDE (BDE-47, BDE-99, BDE-100, and BDE-153) at delivery (B).


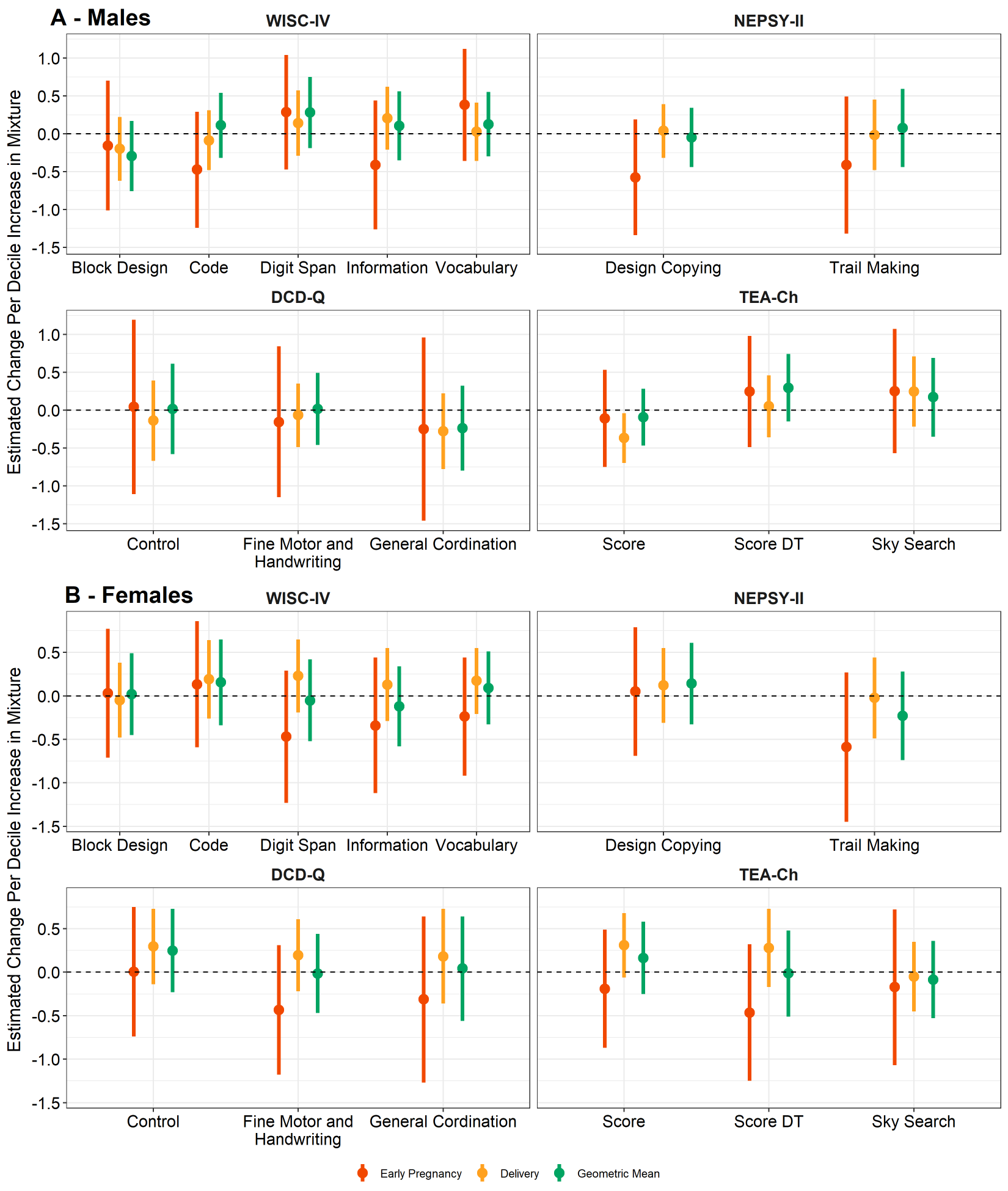


**Figure S3.** Forest plots showing effect estimates (β) and 95% confidence intervals of the association between child neurodevelopmental measures and plasma PBDE concentrations at early pregnancy, delivery, or their geometric mean, stratified by A) Male and B) Female children. The effect estimates are interpreted as the change in test scores per decile increase in all four congeners (BDE-47, BDE-99, BDE-100, and BDE-153). All models were adjusted for maternal age, smoking status during pregnancy, BMI, and total plasma lipids.
